# A Debilitating Orthopaedic Complication following Corticosteroid Therapy for Polymyalgia Rheumatica

**DOI:** 10.1155/2014/515361

**Published:** 2014-04-06

**Authors:** Paul Rai, Vinay Takwale

**Affiliations:** ^1^Cheltenham General Hospital, Sandford Road, Cheltenham, Gloucestershire GL53 7AN, UK; ^2^Gloucestershire NHS Trust, UK

## Abstract

Avascular necrosis (AVN) of the scaphoid secondary to corticosteroid use is a rare entity. Previous reports in the literature refer to chronic steroid intake. We report a case secondary to low dose, short term use. AVN has a multifactorial cellular and genetic aetiology and most frequently affects the femoral head. Diagnosis relies on a high index of suspicion and early magnetic resonance (MR) scanning. Treatment options are similar to those of traumatic scaphoid nonunions and include vascularised bone grafting and scaphoid excision. Polymyalgia Rheumatica is a common condition and its treatment is led by corticosteroid use. Mild to moderate strengths are advocated. However in our report we show that even with small doses serious adverse effects can be encountered.

## 1. Introduction

### 1.1. Nontraumatic Avascular Necrosis

Avascular necrosis (AVN) is a condition that occurs at multiple sites in the body with vulnerable arterial perfusion. There are traumatic and nontraumatic causes. Avascular necrosis of the scaphoid in the absence of trauma is a rare but recognised condition. There are many known associated risk factors and precipitants of AVN. The major categories include drugs (most commonly corticosteroids [1]), infection, coagulation disorders, haemoglobinopathies, and miscellaneous causes. The common miscellaneous causes are Perthes' disease, alcohol, systemic lupus erythematosus (SLE), and pregnancy [2].

Corticosteroids are thought to induce avascular necrosis by two mechanisms: (1) intravascular thrombosis of sinusoids and capillaries in the bone marrow and (2) increasing marrow pressure, reducing intraosseous perfusion. Sinusoidal thrombosis results in vascular stasis increasing arterial occlusion further in a vicious cycle [2].

The clinical features usually consist of an initial asymptomatic period, followed by worsening pain and swelling. Restricted movement and deformity may be encountered. The radiograph changes are minimal in the first few months. Sclerosis from attempts at new bone formation appears after this time with preservation of the joint line. Late in the disease, joint line destruction and deformity become apparent. With the recent increased availability of MR scanning, changes can be seen a lot sooner with areas of low intensity signal on T1 and T2 weighted images early in the disease [3].

### 1.2. Polymyalgia Rheumatica (PMR)

PMR is the most common inflammatory rheumatological condition among elderly people in the United Kingdom (UK). It has an estimated prevalence of 2% in people over 60 years of age in the UK [4]. The British Society of Rheumatology (BSR) guidelines have a clear algorithm for starting such patients on corticosteroid treatment [5]. The doses are low to moderate strength; they are variable in their length of prescription and depend on symptom response. The myriad of side effects and adverse events associated with steroid therapy are widely documented and not discounted by the BSR. They advise clinicians to taper down treatment as soon as an appropriate positive response has been achieved.

## 2. The Case

A 69 year old right hand dominant lady presented in January 2012 with a 3-week history of spontaneous pain and swelling on the radio-volar aspect of her left wrist. She is a retired nursing manager and had a past medical history of Polymyalgia Rheumatica, breast carcinoma, osteoporosis, and ischaemic heart disease. Her PMR was diagnosed by her GP in October 2011 when she began a two-month course of Prednisolone at a dose of 20 mg per day. Her breast malignancy was treated with lumpectomy and tamoxifen 20 years ago.

**Figure 1:**
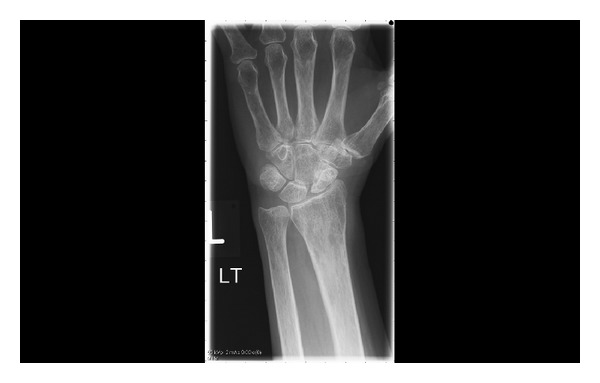
Plain AP radiograph of the carpus.

On examination of the wrist, the swelling was warm to touch and she had a range of movement of 60 degrees (flexion/extension). Radiographs at the time revealed periarticular osteoporosis and no other abnormalities (Figure 1).

**Figure 2:**
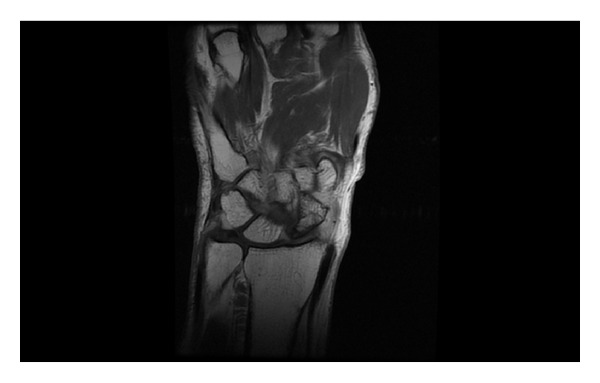
Coronal slice of MRI showing carpus.

An MR scan at 4 weeks after presentation (Figure 2) was reported to be showing acute synovitis of the wrist joint and sclerosis of the proximal pole of the scaphoid consistent with avascular necrosis.

At four months she developed paraesthesia and worsening pain in the same area of the wrist. She was managing with nonsteroidal analgesia, although household tasks were difficult and she was unable to drive her car.

Her case was discussed at a regional hand surgeons conference, where difficult local cases are discussed. As a result of this meeting, avascular necrosis secondary to corticosteroid therapy was the agreed working diagnosis. There were no features of Giant Cell Arteritis in this case. Management options discussed in the meeting included proximal row carpectomy and scaphoid excision plus four-corner fusion.

Proximal row carpectomy involves an incision and removal of the scaphoid, lunate, and triquetrum bones [6]. Four-corner fusion involves the removal of the scaphoid and internal fusion of the lunate, triquetrum, capitate and hamate [7]. The treating team opted for the former. This lady was listed for theatre in April. However she sought a second opinion and underwent an arthroscopy in July. The arthroscopy revealed an abnormally soft scaphoid, again consistent with avascular necrosis. She underwent a proximal row carpectomy in August, nine months after the onset of symptoms. Since her operation she has been progressing well with physiotherapy. At five months postoperatively she was contacted by telephone. Her pain and function were improved. Household jobs and driving were manageable again. Incidentally she did not require any further treatment for her PMR.

## 3. Discussion

The most common cause of nontraumatic avascular necrosis is corticosteroid use [1]. Recent reviews have suggested the mechanism of steroid-induced avascular necrosis to be multifactorial and complex. Steroids affect osteoblast differentiation, osteoclast apoptosis, and calcium and lipid metabolism [8]. Host factors and genetics are likely to be involved as well [1].

There are a vast amount of studies referring to steroid induced avascular necrosis. The most common site to be affected is the femoral head.

Li et al. reviewed 539 SARS patients on corticosteroids and found 32.7% with evidence of avascular necrosis somewhere in the body after one month. The study was performed in China and included doses of steroid ranging from 80 mg to 30 G/day. They also found 11 cases of scaphoid or lunate AVN [9].

Nontraumatic avascular necrosis of the scaphoid was first described by Preiser in 1910 [10]. However it has since been found that he falsely described the disease, as his case is now thought to have been secondary to trauma. There are many references to idiopathic AVN of the scaphoid and two reports of the condition secondary to corticosteroid use in particular [11, 12]. Both were associated with long-term use. Our case was presumed to be secondary to steroid use as the events were temporally related. Therefore screening for other aetiologies was not performed. If the aetiology is in doubt, testing for haemoglobinopathies and coagulation disorders should be considered.

The timing of diagnosis in AVN is crucial and if delayed will increase the likelihood of irreversible morphological bony changes associated with fracture of subchondral trabeculae. MRI is recognized as the most sensitive imaging modality [13]. One study suggested an MRI screening programme for all patients taking corticosteroids [1]. Other possible modalities are Computed Tomography (CT), single-photon emission CT scanning, and nuclear imaging. However, none have been shown to be as sensitive as MRI. The use of Ultrasound is increasing in PMR, but it is likely to only show features of advanced AVN [14].

The route of administration and dose of corticosteroids are also important. Powell et al. found that cases were mostly attributed to high dose systemic use [8]. Li et al. clearly demonstrate the effects of high doses on AVN rates in their group of SARS patients [9]. In our case the patient developed avascular necrosis of the scaphoid secondary to low dose, systemic steroid use and only after two months.

The treatment options for nontraumatic AVN of the scaphoid are similar to those for difficult scaphoid nonunions secondary to trauma. The options are broadly: bone grafting procedures or palliative surgical procedures. Most recently, vascularised pedicle bone grafting has shown good results in halting and even reversing avascular necrosis in the scaphoid [15–17]. For cases which are advanced and not amenable to grafting (i.e., stage III AVN in nonunions) palliative surgical options are: proximal row carpectomy or scaphoid excision plus four-corner fusion [13]. There are no substantial reviews of the efficacy of these management options, but vascularised bone grafting has shown good results in early disease in three recent papers concerned with the scaphoid for relieving pain [15–17].

Although nontraumatic avascular necrosis is likely to be a rare occurrence in low dose corticosteroid use, the consequence, for our patient, who initially presented with proximal muscle pain (PMR), were detrimental. She suffered a debilitating wrist condition and her options for relief involved radical surgery to an important functional area of the body.

## 4. Conclusions

It is important when treating rheumatological conditions, such as PMR, to recognise the potential side effects of corticosteroid therapy. The common effects are well known, but avascular necrosis should be suspected in those with new bony pain. As shown here it can occur after even short courses of steroids. If suspected, refer promptly for investigation and withhold treatment.
